# Fracture of the Lower Jaw

**Published:** 1859-08

**Authors:** 


### FRACTURE OF THE LOWER JAW.

In this case there was unusual difficulty in maintaining the fragments in apposition, by any means or appliances in ordinary use, but in which accurate union was at length obtained by an apparatus made by Mr. Howard Hayward, dentist. This consisted of a silver plate, moulded so as to fit accurately upon the teeth, and taking hold of three or four teeth on each side of the fracture. From this plate two pieces of metal projected at the angles of the mouth, and were tied by string to a small piece of pasteboard placed under the chin. Thus was the immobility of the fracture secured, and the patient relieved from a condition of extreme distress, and enabled without pain to open and close his mouth for the purpose of taking food, and also to articulate with tolerable clearness.—*Ib.*

